# Optimal Scan Time of ^18^F-FDG PET in Identifying Therapeutic Efficacy Secondary to Radiofrequency Ablation of Lung Cancer: Case Report

**DOI:** 10.1097/MD.0000000000000884

**Published:** 2015-06-19

**Authors:** Wen-Qian Zhang, Yi-Li Fu, Bin Hu, Shuo Chen, Min-Fu Yang, Hui Li

**Affiliations:** From the Department of Thoracic surgery (W-QZ, Y-LF, BH, SC, HL), Beijing Institute of Respiratory Diseases, Beijing Chaoyang Hospital; and Department of Nuclear Medicine (M-FY), Chaoyang Hospital, Capital Medical University, Beijing, China.

## Abstract

Positron emission tomography (PET)/computed tomography (CT)–guided radiofrequency ablation (RFA) has become a major treatment approach for small tumors. Identifying this quantitative dynamic ^18^F-2-fluoro-2-deoxy-d-glucose (^18^F-FDG) activity at the primary lesion can minimize misdiagnosis and allow an opportunity to reintervene.

Here, we report 3 patients with nonsmall cell lung cancer (NSCLC) who underwent the ablative therapy with split-dose ^18^F-FDG fused PET/CT scans for early identification of residual tumors and follow-up evaluation of treatment.

Our results indicate that reliable post-RFA imaging follow-up is critical in fast and efficient assessment of complete tumor resection in patients experienced the ablation procedure.

## INTRODUCTION

Nonsmall cell lung cancer (NSCLC) is the most common type of lung cancer worldwide. Surgical resection of the cancer is the primary treatment since it is relatively insensitive to chemotherapy.^1,2^ However, only one-fifth of the patients are surgical candidates due to their advanced stage at presentation or medical comorbidities. Radiofrequency ablation (RFA) has received considerable attention, mainly for relatively small tumors ^3–5^ and local therapy that may benefit those of the patients.^6–8^ So far, a major challenge of RFA therapy has been reliable postprocedural assessment for its early therapeutic efficacy.

A quantitative dynamic ^18^F-2-fluoro-2-deoxy-d-glucose (^18^F-FDG) positron emission tomography (PET) scan is increasingly being used in the initial diagnosis and follow-up posttherapy for a wide variety of cancer patients.^9,10^ Since NSCLC has a more dismal prognosis generally with widespread disease that is not amenable to surgical resection, an earlier prediction of whether RFA benefits the cancer patients provided by ^18^F-FDG PET imaging can guide doctors in offering them better care. This study is conducted to demonstrate that repeatable ^18^F-FDG PET/CT (computed tomography) scans may characterize an early therapeutic response to RFA and provide endpoint evaluation for postprocedural treatment of the NSCLC patients.

## METHODS

### Study Patients

Three patients with NSCLC underwent RFA with split-dose ^18^F-FDG PET/CT scans during a period of 4 months from August to November 2013. The diagnosis of NSCLC was established according to the pathological results by either CT-guided transthoracic needle biopsy or bronchoscopic biopsy from the lung lesions. All patients did not have diabetic history. This study was approved by the Institutional Ethics Committee of Beijing Chaoyang Hospital of Capital Medical University; the Ethics Committee also approved the related scanning, RFA therapy, and data collection from these patients based on the analysis of clinical outcome. All subjects signed written informed consent forms for this study.

### PET/CT-Guided RFA Procedure

**FIGURE 1:**
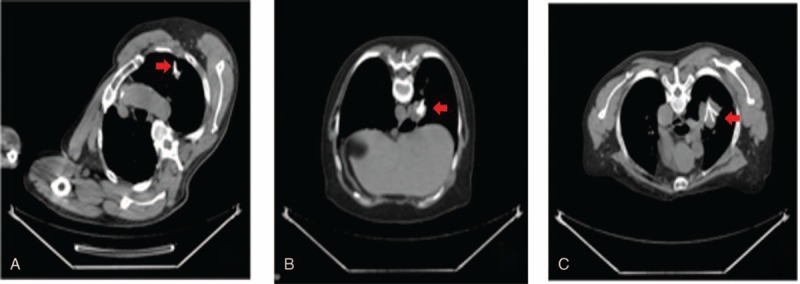
Placement of radiofrequency electrode was observed by a tiny, needle-like probe inserted to lung cancer tissues under CT guidance. Pretreatment CT scanning in patient 1 (A), 2 (B), and 3 (C) showed an active tip (red arrow) of a needle electrode within the targeted lesion. CT = computed tomography.

Percutaneous CT-guided RFA procedure was carried out using an initial dose of 4 mCi of ^18^F-FDG injected to each patient. A single-needle electrode for radiofrequency tissue ablation was placed within the lesion tissue and was visualized in the FDG-avid regions (Figure 1A–C). The procedure was implemented in the patients under local anesthesia. We used an automatic apparatus with maximal power output of 150 W operating at 460 Hz (Model 1500; RITA Medical System, Mountain View, CA). It has multiple temperature displays and power monitoring, and software is available to record and graphically represent all the data on a personal computer. Two grounding pads were applied to each shaved leg to ground the current and to reduce risks of skin thermal injury. The tumor tissue was ablated at 90°C for 15 to 27 minutes according to the size of the tumor. Vital signs are continuously, noninvasively monitored. The entire procedure was completed within 64 to 70 minutes.

### Approach for ^18^F-FDG PET Data Acquisition

All patients were instructed to fast for at least 6 hours before ^18^F-FDG PET imaging and then each patient received a preoperative injection of ^18^F-FDG on the same day as surgery. A dose of 4 mCi (148 MBq) ^18^F-FDG was administered intravenously into a peripheral vein of each patient prior to the anticipated time of surgery. A PET/CT image was acquired 60 minutes after the radioactive tracer injection using a GE Discovery STE 16 PET/CT scanner (Healthcare, Milwaukee, WI). The patients were positioned in the scanner using laser guides aligned to the lung lesion and confirmed by a CT scout scan. PET imaging was immediately preceded by transmission CT for attenuation correction and anatomic correlation purposes. Once the preoperative clinical PET/CT scans were acquired, images were reviewed by the oncologic surgeon and the nuclear medicine physician.

Postoperatively, each patient was injected by an additional dose of 8 mCi (296 MBq) ^18^F-FDG and followed with a PET/CT scan reimaged at 45 minutes after the completion of the PET/CT tumor ablation procedure. The postoperative PET/CT scan allowed for reverification of completeness of tumor destruction and an opportunity for repeat intervention in the residual lesion while patients were still on the table. All patients were also followed up with the ^18^F-FDG PET/CT-based metabolic imaging for endpoint evaluation of therapeutic response to the tumor ablation in a 3-month interval after RFA.

### Measurement of Maximum Standardized Uptake Value

Semiquantitative estimation of tumor glucose metabolism by use of standardized uptake value (SUV) is based on relative lesion radioactivity on images corrected for attenuation and normalized for the injected dose and body surface area. A maximum SUV from normal tissue (SUVmax-NT) and lesion tissues (SUVmax-LT) was acquired within a PET image coregistered with a CT image. SUVmax derived from the method was computed for ^18^F-FDG uptake quantitation in the cancer tissue and an average value of the SUVmax measurements was calculated through drawing 3 similar spheres of interest within the same ^18^F-FDG PET image in each patient. Following SUVmax measurements in the field, a ratio of SUVmax-LT/SUVmax-NT was calculated within the same PET image.

### Statistical Analysis

Values were expressed as mean ± standard deviation (SD) on some of the results from the investigated patients. Statistical analysis was performed using Statistical Package for the Social Science (SPSS, version 22.0). Comparisons from SUVmax measurements and its ratios were performed by Student paired test in between of 2 patients. A *P* value of <0.05 was considered significant.

## RESULTS AND DISCUSSION

### Results

**FIGURE 2:**
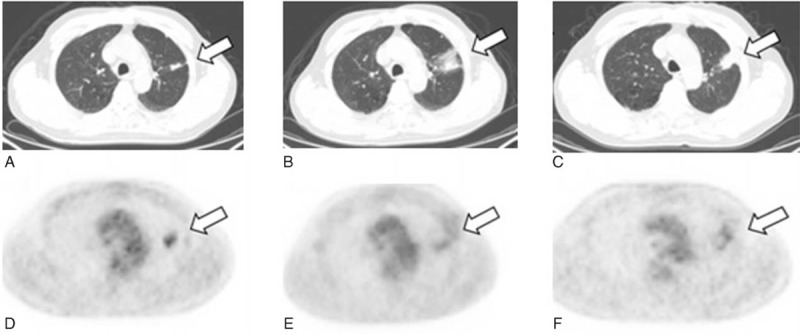
PET/CT-guided RFA applied for a 60-year-old male patient with NSCLC (squamous cell carcinoma of 1.9 × 0.7 cm in size). Split dose ^18^F-FDG methodology included pretreatment with 4 mCi of FDG followed by a postprocedure dose of 8 mCi for same-site treatment assessment. In comparison with a preoperative ^18^F-FDG PET/CT imaging (A, D), the size and metabolic state of the residual tumor were accurately characterized with split-dose ^18^F-FDG PET/CT reimaged at 45 minutes (B, E) and 3 months (C, F) after RFA respectively. The images revealed a focal ^18^F-FDG activity in the ablation zone at 45 minutes and 3 months after the treatment. ^18^F-FDG = ^18^F-2-fluoro-2-deoxy-d-glucose, NSCLC = nonsmall cell lung cancer, PET/CT =  positron emission tomography/computed tomography, RFA =  radiofrequency ablation.

**FIGURE 3:**
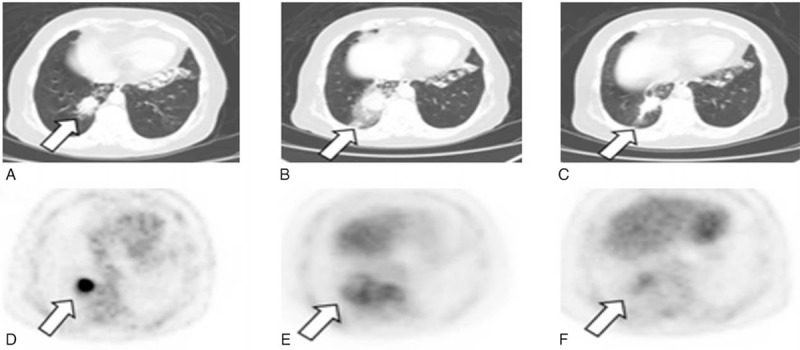
A change in SUVmax-LT (A) in the ablated regions and a ratio (B) of SUVmax-LT/SUVmax-NL were examined in the NSCLC patients. A ratio in the second ^18^F-FDG PET scan was shown as 2.094 and 2.02 in case 1 and 2 and 3.834 in case 3, respectively. Data were expressed as mean ± SD (3 measurements). ^18^F-FDG  = ^18^F-2-fluoro-2-deoxy-d-glucose, PET = positron emission tomography, NSCLC = nonsmall cell lung cancer, RFA =  radiofrequency ablation, SUVmax-LT =  maximum SUV from lesion tissues, SUVmax-NT =  maximum SUV from normal tissue. ∗ *P* < 0.01 versus another 2 cases at the same time points.

Case 1: A 60-year-old male was admitted to our hospital because of cough and expectoration for 3 months. He was diagnosed as lung lesion by a chest x-ray test and associated with lung function decline reaching to 51.4% of predicted normal values for forced expiratory volume in 1 second (FEV_1.0_). A CT scan before RFA showed a high-density mass of 1.9 × 0.7 cm in the apical segment of the left upper lobe (Figure 2A). The specimen from the lesion tissue was taken by CT-guided transthoracic needle biopsy and the pathological result revealed stage IIIA squamous cell carcinoma. In association with the preoperative result of CT, an initial dose of 4-mCi of ^18^F-FDG for PET/CT imaging was administered intravenously into a peripheral vein of the patient and the PET imaging displayed a hypermetabolic state of ^18^F-FDG uptake in the region of lung lesion (Figure 2D). After the patient underwent the image-guided RFA under conscious sedation, an additional dose of 8 mCi ^18^F-FDG applied for PET/CT scanning was performed 45 minutes after RFA and the same dose of ^18^F-FDG-based imaging was done 3 months after the tumor ablation (Figure 2B, C, E, F), respectively. ^18^F-FDG PET/CT features were suggestive of peripheral ground glass opacities obscuring and surrounding the tumor ablation zone with heterogeneous density in the ablated zone at the indicated times. SUVmax measurements in the ablation zone expressed an increased ^18^F-FDG uptake as compared to that before RFA (Figure 3A). However, a ratio of SUVmax-LT/SUVmax-NT in the ablated region was significantly decreased to 2.094 (45 minutes) and 2.048 (3 months) from 3.93 before RFA (Figure 3B).

**FIGURE 4:**
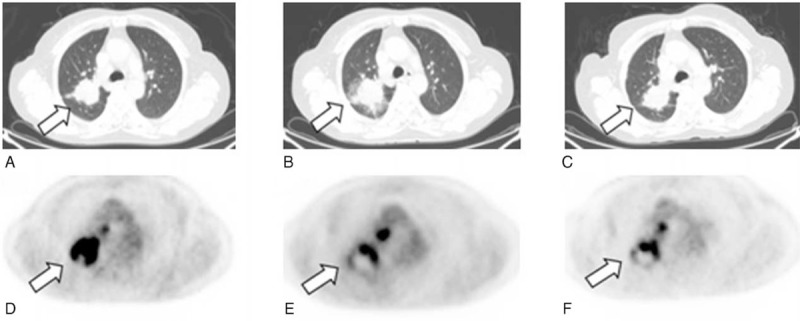
A 57-year-old woman with NSCLC (adenocarcinoma of 3.1 × 2.2 cm in size) received the PET/CT-guided RFA. Split-dose ^18^F-FDG methodology included pretreatment with 4 mCi of FDG followed by a postprocedure dose of 8 mCi for same-site treatment assessment. The size and metabolic state of the residual tumor were accurately determined in ^18^F-FDG PET/CT scans before RFA (A, D), and 45 minutes (B, E) and 3 months (C, F) after the treatment, respectively. The images revealed a focal ^18^F-FDG activity in the ablation zone at 45 minutes and 3 months after the treatment.^ 18^F-FDG = ^18^F-2-fluoro-2-deoxy-d-glucose, NSCLC = nonsmall cell lung cancer, PET/CT = positron emission tomography/computed tomography, RFA =  radiofrequency ablation.

Case 2: A 57-year-old woman was admitted to our hospital because of cough chest tightness and shortness of breath for 1 month and diagnosed as lung lesion by a chest x-ray test with lung function decline reaching to 30.5% of predicted normal values for FEV_1.0_. In association with CT examination, a high-density mass at 3.1 × 2.2 cm was detected at the medial base of the right lung (Figure 4A). The specimen from the mass was taken by bronchoscopic biopsy and the pathological result of the lung lesion revealed stage IIA adenocarcinoma. The patient likewise received an initial dose of 4-mCi of ^18^F-FDG for PE/CT scanning prior to RFA and the result showed an increased ^18^F-FDG uptake in the lung lesion zone (Figure 4D). After the patient experienced the PET/CT-guided RFA under local anesthesia, a spilt-dose of 8 mCi of ^18^F-FDG for PET/CT scanning applied for monitoring its metabolic activity in the ablation zone 45 minutes and 3 months after RFA (Figure 4 B, C, E, F). The ^18^F-FDG PET/CT scan likewise showed the ablation lesion was ground glass opacities with blurry margin and had heterogeneous density. SUVmax measurement after RFA displayed an increased ^18^F-FDG uptake within the ablation area as compared to that before RFA (Figure 3A). In contrast, a ratio of SUVmax-LT/SUVmax-NT in the ablated zone was significantly decreased to 2.02 (45 minutes) and 3.628 (3 months) from 3.864 before the RFA procedure (Figure 3B).

**FIGURE 5:**
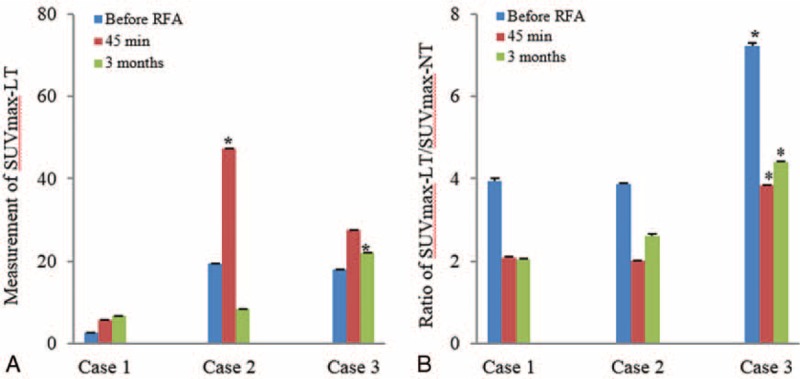
A 59-year-old woman with NSCLC (adenocarcinoma of 3.8 × 3.5 cm in size) underwent the PET/CT-guided RFA. Split-dose ^18^F-FDG methodology included a localization dose of 4-mCi FDG followed by a postprocedure dose of 8 mCi for same-site treatment assessment. In contrast to the increased ^18^F-FDG uptake in the regional tumor before the RFA procedure (A, D), the ^18^F-FDG activity remained in the tumor ablation zone may be detected in ^18^F-FDG PET/CT scans reimaged at 45 minutes (B, E) and 3 months (C, F) after RFA, respectively. ^18^F-FDG = ^18^F-2-fluoro-2-deoxy-d-glucose, NSCLC = nonsmall cell lung cancer, PET/CT = positron emission tomography/computed tomography, RFA =  radiofrequency ablation.

Case 3: A 59-year-old woman presented with cough with bloody sputum for 1 month and diagnosed as lung mass on a chest x-ray with lung function decline showing 66.7% of normal predicted FEV_1.0_. CT scan displayed a high-density mass of 3.8 × 3.5 cm in the right upper lobe posterior segments (Figure 4A). The specimen from the mass tissue was obtained by bronchoscopic biopsy and the pathological result indicated stage IV adenocarcinoma. The patient received the image-guided RFA under local anesthesia with a spilt-dose of 8 mCi ^18^F-FDG PET/CT scanning for evaluating a metabolic state in the ablation zone 45 minutes and 3 months after the treatment (Figure 5B, C, E, F). The results of ^18^F-FDG PET/CT scans revealed ground glass opacities surrounding the ablated region with the same density remained in the region. SUVmax measurement exhibited intensive ^18^F-FDG uptake within residual cancer tissues at the indicated times (Figure 3A). However, the ratio of SUVmax-LT/SUVmax-NT in the ablated area was decreased to 3.834 (45 minutes) and 4.386 (3 months) from 7.232 of the pretreatment (Figure 3B). In contrast, we found that case 1 and case 3 showed a significant decrease in SUVmax-LT as compared to case 2 at 45 minutes but not 3 months after RFA (both *P* < 0.01). In further observation, there were statistical differences detected in the reduced ratio at 45 minutes and 3 months after RFA between case 3 and either case 1 or case 2 (both *P* < 0.01). Since the residual cancer was closed to precava and the right pulmonary artery, reablation for the patient was not executed in the probe ablative therapy to avoid vascular injury.

## DISCUSSION

NSCLC is the most common cause of cancer death worldwide and the suggested first-line treatment for early-stage NSCLC is surgical resection.^11^ Unfortunately, some patients are considered inoperable because systemic failures can be problematic even in early-stage disease. RFA shows a high merit in an alternative treatment for pulmonary malignancies because this technique may offer faster, more targeted lung cancer treatment with fewer side effects compared with standard therapies.^12–14^ Furthermore, it has some advantages over traditional radiotherapy and chemotherapy based on the fact that its safety profile is similar to percutaneous image-guided lung biopsy. In this study, we reported our results in treating 3 NSCLC patients with RFA by using repeatable ^18^F-FDG PET/CT imaging for early recognition of residual tumors and follow-up evaluation of the treatment.

In case 1 and 2, treatment with RFA resulted in an increased ^18^F-FDG uptake and peripheral ground glass opacities surrounding the tumor ablation zone in ^18^F-FDG PET images during the period of 45 minutes to 3 months after RFA. Semiquantitative estimation of the tumor glucose metabolism showed widely irregular changes in SUVmax-LT, a routine measurement considered as an intention of achieving definitive therapy.^15^ The changes were probably related to surgical procedure and inappropriate measuring manner provided for evaluation of the treatment. Since the increased FDG activity occurred in a short time, our postulate was that it did not refer to an inflammatory response in the targeted lesion tissues to mechanical stimuli by RFA probe. In this RFA procedure, a 0.8-cm margin of apparently healthy tissue at the periphery of the tumor was ablated to reduce the risk of incomplete ablation.^16,17^ The probe-induced damage may causally be considered as a factor relating to the increased activity. Additionally, an extra dose of FDG injected to the postoperative patients with the initial dose of the tracer accumulated in the ablation zone would be an additive factor accounted for the increased uptake in the patients since ^18^F-FDG is metabolized with a radioactivity elimination half-life of 110 minutes.^18^ Based on the above considerations, it was reasonable to speculate that SUVmax was not a sensitive parameter in identifying early efficacy of the post-RFA. In addition to SUVmax measurements, the ratios of SUVmax-LT/SUVmax-NT detected from both patients significantly reduced to 2.09 and 2.02 in the ablated zones 45 minutes after RFA, suggesting that the ratio was involved in the event. In further observation, the reduced ratio was caused mainly due to a small-scale increase in SUVmax-LT as compared to the increase in SUVmax-NT in the same ^18^F-FDG PET oncologic image, indicating that the ratio measured by the second ^18^F-FDG PET imaging was valuable for fast assessment of therapeutic response to RFA. Such, it is likely that the ratio test at the indicated time was not only effective but also superior to SUVmax in early evaluation of therapeutic efficiency of the post-RFA. In support of the consideration, the reduced ratio was verified with a relatively stable change remaining until 3 months, demonstrating such a particular importance of the ratio in charactering early efficacy of the RFA procedure and providing an endpoint assessment for the therapeutic efficiency in the residual lesion in 3 months after RFA. Since the individual values of the ratio obtained from both patients were extremely similar in the second scan, it is reasonable to suggest that the reduced ratio in the ablated zone 45 minutes after RFA would be an important indicator to validate immediate efficacy of the postprocedural treatment.

In case 3, split-dose ^18^F-FDG PET imaging displayed the ablation lesions that were ground glass opacities with blurry margin and had higher density in the ablated zone 45 minutes and 3 months after RFA. In contrast to case 1 and 2, SUVmax was also significantly increased in the tumor ablation region, but a relatively high ratio of 3.83 was detected at 45 minutes and 4.39 in 3 months after RFA, suggesting that the patient failed to the ablation therapy because of an insufficient resection of the lung tumor. This result of the patient with RFA also supported our previous consideration on the importance of the ratio measured by the second ^18^F-FDG PET scan. The incompletion of the tumor ablation occurred probably due to the tumor diameter of > 3.5 cm based on the clinical researches that RFA technique mainly applied for some small lung tumors.^4,19,20^ Due to the tumor ablated near to precava and right pulmonary artery, the patient was not reablated for avoiding vascular injury. Since a comparable success rate of RFA is involved in the treatment of NSCLC patients, it is conceivable that patient selection and preprocedural evaluation for the application of RFA is a key to the successful treatment of the patients, so the selection process takes into consideration a number of factors, including the tumor size and location in lungs, and ultimately becomes a clinical judgment depending largely on surgeon experience.

Collectively, this study highlights the potential importance of the second ^18^F-FDG PET oncologic image in which the ratio of SUVmax measurement would be valuable in identifying an immediate efficacy of tumor ablation and providing fast evaluation of residual malignancy. Future studies are required for quantitative analysis of ratio values in a large cohort of the patients with RFA.

## References

[1] Korst RJ Ginsberg RJ Appropriate surgical treatment of resectable non-small-cell lung cancer. *World J Surg* 2001; 25:184–188. 11338020 10.1007/s002680020017

[2] Scott WJ Howington J Feigenberg S American College of Chest Physicians Treatment of non-small cell lung cancer stage I and stage II: ACCP evidence-based clinical practice guidelines (2nd edition). *Chest* 2007; 132 (3 suppl):234S–242S. 17873171 10.1378/chest.07-1378

[3] Chua TC Sarkar A Saxena A Long-term outcome of image-guided percutaneous radiofrequency ablation of lung metastases: an open-labeled prospective trial of 148 patients. *Ann Oncol* 2010; 21:2017–2022. 20335366 10.1093/annonc/mdq098

[4] Gillams AR Lees WR Radiofrequency ablation of lung metastases: factors influencing success. *Eur Radiol* 2008; 18:672–677. 18008074 10.1007/s00330-007-0811-y

[5] Lee JM Jin GY Goldberg SN Percutaneous radiofrequency ablation for inoperable non–small lung cancer and metastasis: preliminary results. *Radiology* 2004; 230:125–134. 14645875 10.1148/radiol.2301020934

[6] Nguyen CL Scott WJ Young NA “Radiofrequency ablation of primary lung cancer: results from an ablate and resect pilot study. *Chest* 2005; 128:3507–3511. 16304306 10.1378/chest.128.5.3507

[7] Lanuti M Sharma A Willers H Radiofrequency ablation for stage I non-small cell lung cancer: management of locoregional recurrence. *Ann Thorac Surg* 2012; 93:921–927. 22296982 10.1016/j.athoracsur.2011.11.043

[8] Ambrogi MC Fanucchi O Cionietal R Long-term results of radiofrequency ablation treatment of stage I non-small cell lung cancer: a prospective intention-to-treat study. *J Thorac Oncol* 2011; 6:2044–2051. 22052222 10.1097/JTO.0b013e31822d538d

[9] Dimitrakopoulou-Strauss A Pan L Strauss LG Quantitative approaches of dynamic FDG-PET and PET/CT studies (dPET/CT) for the evaluation of oncological patients. *Cancer Imaging* 2012; 12:283–289. 23033440 10.1102/1470-7330.2012.0033 PMC3485644

[10] Bhatt G Li XF Jain A The normal variant 18F FDG uptake in the lower thoracic spinal cord segments in cancer patients without CNS malignancy. *Am J Nucl Med Mol Imaging* 2013; 3:317–325. 23901357 PMC3715776

[11] Jemal A Siegel R Xu J Cancer statistics, 2010. *CA Cancer J Clin* 2010; 60:277–300. 20610543 10.3322/caac.20073

[12] Fernando HC Radiofrequency ablation to treat non-small cell lung cancer and pulmonary metastases. *Ann Thorac Surg* 2008; 85:S780–S784. 18222217 10.1016/j.athoracsur.2007.11.063

[13] Dupuy DE Zagoria RJ Akerley W Percutaneous radiofrequency ablation of malignancies in the lung. *AJR Am J Roentgenol* 2000; 174:57–59. 10628454 10.2214/ajr.174.1.1740057

[14] Suh RD Wallace AB Sheehan RE Unresectable pulmonary malignancies: CT-guided percutaneous radiofrequency ablation—preliminary results. *Radiology* 2003; 229:821–829. 14657317 10.1148/radiol.2293021756

[15] Berghmans T Dusart M Paesmans M Primary tumor standardized uptake value (SUVmax) measured on fluorodeoxyglucose positron emission tomography (FDG-PET) is of prognostic value for survival in non-small cell lung cancer (NSCLC): a systematic review and meta-analysis (MA) by the European Lung Cancer Working Party for the IASLC Lung Cancer Staging Project. *J Thorac Oncol* 2008; 3:6–12. 18166834 10.1097/JTO.0b013e31815e6d6b

[16] Yamamoto A Nakamura K Matsuoka T Radiofrequency ablation in a porcine lung model: correlation between CT and histopathologic findings. *AJR Am J Roentgenol* 2005; 185:1299–1306. 16247153 10.2214/AJR.04.0968

[17] Giraud P Antoine M Larrouy A Evaluation of microscopic tumor extension in non-small-cell lung cancer for three-dimensional conformal radiotherapy planning. *Int J Radiat Oncol Biol Phys* 2000; 48:1015–1024. 11072158 10.1016/s0360-3016(00)00750-1

[18] Miele E Spinelli GP Tomao F Positron Emission Tomography (PET) radiotracers in oncology—utility of 18F-Fluoro-deoxy-glucose (FDG)-PET in the management of patients with non-small-cell lung cancer (NSCLC). *J Exp Clin Canc Res* 2008; 27:52. 10.1186/1756-9966-27-52 PMC2579910 18928537

[19] Bargellini I Bozzi E Cioni R Radiofrequency ablation of lung tumours. *Insights Imaging* 2011; 2:567–576. 22347976 10.1007/s13244-011-0110-7 PMC3259330

[20] Anderson EM Lees WR Gillams AR Early indicators of treatment success after percutaneous radiofrequency of pulmonary tumors. *Cardiovasc Intervent Radiol* 2009; 32:478–483. 19127381 10.1007/s00270-008-9482-6

